# Value of Fluorodeoxyglucose Positron Emission Tomography/Computed Tomography in Identifying Osteoarticular Septic Grafts in Suspected Infective Endocarditis: Results from a Large Monocentric Cohort

**DOI:** 10.3390/jcm13185419

**Published:** 2024-09-12

**Authors:** Cédric Luczak, Lionel Lerman, Laura Pina Vegas, Berivan Emsen, Benjamin Hugues, Raphaël Lepeule, Julien Ternacle, Raphaëlle Huguet, Pascal Lim, Jean-Winoc Decousser, Antonio Fiore, Emmanuel Itti, Xavier Chevalier, Mukedaisi Abilizi, Florent Eymard

**Affiliations:** 1 Department of Rheumatology, Assistance Publique—Hôpitaux de Paris, Henri Mondor Hospital, 94000 Créteil, France; cedric.luczak@aphp.fr (C.L.); laura.pinavegas@aphp.fr (L.P.V.); hugues.benjamin@proton.me (B.H.); xavier.chevalier@aphp.fr (X.C.); 2 Department of Nuclear Medicine, Assistance Publique—Hôpitaux de Paris, Henri Mondor Hospital, 94000 Créteil, France; lionel.lerman@aphp.fr (L.L.); berivan.emsen@gmail.com (B.E.); emmanuel.itti@aphp.fr (E.I.); amukeddes@yahoo.fr (M.A.); 3 Department of Microbiology, Assistance Publique—Hôpitaux de Paris, Henri Mondor Hospital, 94000 Créteil, France; raphael.lepeule@aphp.fr (R.L.); jean-winoc.decousser@aphp.fr (J.-W.D.); 4 Department of Cardiology, Haut Leveque Hospital, 33600 Pessac, France; julien.ternacle.hmn@gmail.com; 5 Department of Cardiology, Assistance Publique—Hôpitaux de Paris, Henri Mondor Hospital, 94000 Créteil, France; raphaelle.huguet@aphp.fr (R.H.); pascal.lim@aphp.fr (P.L.); 6 Department of Cardiac Surgery, Assistance Publique—Hôpitaux de Paris, Henri Mondor Hospital, 94000 Créteil, France; antonio.fiore@aphp.fr

**Keywords:** infective endocarditis, osteo articular septic graft, FDG-PET/CT, tricuspid valve

## Abstract

**Background:** 18F-fluorodeoxyglucose positron emission tomography–CT (FDG-PET/CT) is useful for identifying infective endocarditis (IE) but also the detection of other concomitant septic foci. Previously, we found that FDG-PET/CT identified an osteoarthritic septic graft (OASG) in 19.1% of IE patients, frequently asymptomatic. These preliminary results encouraged us to extend our analyses to a larger population, including all patients initially explored for suspected IE, to assess the prevalence, characteristics, and OASG locations brought out by FDG-PET/CT and to identify predictive factors. **Methods:** From a single-center cohort of patients referred for a clinical and/or biological suspicion of IE, we included all patients who underwent FDG-PET/CT, mainly performed to confirm a prosthesis heart valve or a foreign cardiac device infection. We excluded those who did not meet the 2015 modified Duke Criteria and those for whom another infectious diagnosis was finally retained or for whom all bacterial samples were negative. Demographic, clinical, bacteriological, imaging, and therapeutic data were collected. FDG-PET/CT images were retrospectively analyzed by three blinded nuclear medicine specialists to identify OASGs. **Results:** We identified 72 distinct OASG locations by FDG-PET/CT in 48 of 174 patients (27.6%), mainly located in the spine (21 OASGs in 20 patients); 14 patients (8.0%) had several OASG locations. In total, 43.8% of OASG locations were asymptomatic. In multivariate analysis, the presence of OASGs was associated with musculoskeletal pain (*p* < 0.001) and tricuspid valve involvement (*p* = 0.002). **Conclusions:** FDG-PET/CT is useful for identifying OASGs in patients with suspected IE, especially those with tricuspid IE or musculoskeletal pain. The identification of OASGs could impact antibiotic therapy and would allow adapted orthopedic management to be proposed.

## 1. What Is Already Known on This Topic

- Infective endocarditis (IE) may sometimes be revealed by extra-cardiac manifestations, such as osteoarticular septic grafts (OASGs), which are related to hematogenous bacterial migration.

## 2. What This Study Adds

- Over 25% of patients hospitalized for suspected IE had OASGs according to FDG-PET/CT data, mainly localized in the spine and asymptomatic in almost 50% of cases.
- OASGs were associated with tricuspid infection and musculoskeletal pain. There was also a trend towards an association with sustained bacteremia.

## 3. How This Study Might Affect Research, Practice, or Policy

- Based on the 2023 European Society of Cardiology guidelines suggesting that PET-FDG/CT could be considered for screening peripheral lesions in asymptomatic patients, the findings of this exam could be particularly useful in patients with tricuspid endocarditis or a sustained bacteremia to detect asymptomatic OASGs.

## 4. Introduction

Infective endocarditis (IE) primarily involves heart valves (native or prosthetic). Its annual incidence is estimated at 34 per million in France [1], and the mean age of affected individuals is 62 years. IE is caused by bacteremia related to bacterial inoculation, capillary fragility in the context of digestive cancer, poor oral condition, or immunodeficiency (renal failure, dialysis, Human Immunodeficiency Virus (HIV) infection, cirrhosis, or neoplasia). Its diagnosis is based on the modified Duke criteria, including major and minor criteria, which were updated in 2023 by the International Society for Cardiovascular Infectious Diseases (ISCVID) [2] and the European Society of Cardiology (ESC) [3], and its treatment requires the identification of the causative micro-organism and its eradication by targeted antibiotic therapy, lasting from 2 to 6 weeks. IE remains a serious infection, fatal in 20% to 30% of cases [4].

IE may sometimes be associated with extra-cardiac manifestations related to hematogenous bacterial migration, especially septic grafts in the skin, central nervous system, kidney, and spleen but also muscle, bone, and joints. Osteoarticular septic grafts (OASGs) mainly involve spine and peripheral joints and can sometimes reveal IE [5]. The prevalence of OASGs is estimated at about 15%. In a large cohort, spondylodiscitis accounted for up to 61% of IE-associated musculoskeletal (MSK) infections, whereas axial arthritis (sternoclavicular, pubic symphysis, interapophyseal, or sacroiliac joints) and peripheral arthritis accounted for 21% and 27%, respectively [6]. Most osteoarticular infections should be treated with antibiotics for 4 to 6 weeks [7,8].

18F-fluorodeoxyglucose positron emission tomography–CT (FDG-PET/CT) has a major role in the diagnosis of IE mainly in prosthetic heart valves, with a sensitivity of 73% and specificity of 80%. It was first included in the 2015 Duke–ESC criteria for patients with prosthetic valves, increasing the sensitivity from 70% to 97% [9], and has become a major criterion for all patients in the 2023 updated version [2]. FDG-PET/CT also enables whole-body mapping of hypermetabolism and thus can detect potential secondary septic locations. In recent IE cohorts examined by FDG-PET/CT, the prevalence of spondylodiscitis could reach 20% [10,11].

From a large monocentric cohort, we described for the first time the prevalence and locations of OASGs identified by FDG-PET/CT in 89 patients with a probable or definite IE according to the modified Duke criteria. In total, 23 OASG locations were found in 19.1% of IE patients, mainly in the spine but also in the glenohumeral joint, coxofemoral joint, sternoclavicular joints, and sacroiliac joint. Only 35% of all OASG locations were painful [12].

These preliminary results encouraged us to extend our analyses to a larger population, including all patients with suspected IE, which is closer to initial care in “real life”. Our objectives were to assess the prevalence of OASGs in this population, to describe their characteristics and locations, and to identify their predictors.

## 5. Material and Methods

### 5.1. Population

Our study was based on the “SOS endocarditis” cohort that consecutively included all patients with suspected IE referred to the cardiology intensive care unit of Henri Mondor Hospital (Créteil, France) between July 2015 and December 2020 for a suspected IE based on clinical and/or biological data such as positive blood culture. This cohort study followed the Declaration of Helsinki principles, and all patients gave their written informed consent. The study was approved by the institutional review board of the Assistance Publique-Hôpitaux de Paris (AP-HP), Hôpitaux Universitaires Henri Mondor (CNIL no. 1778041, 28 June 2013).

In our study, we included only patients who underwent FDG-PET/CT, which was mainly performed to confirm a cardiac infection, especially in cases involving a prosthesis heart valve or a foreign cardiac device (pacemaker or implantable cardioverter defibrillator) but also to find another infectious location or to search for neoplasia. We excluded from our analysis patients who did not meet the 2015 Duke–ESC criteria [9] and those for whom another infectious diagnosis was finally retained (e.g., pneumopathy, pyelonephritis, septic arthritis, spondylodiscitis) but also patients for whom all bacteriological samples were negative.

### 5.2. Study Endpoints

The primary endpoint was the prevalence of OASGs based on FDG-PET/CT analysis. Secondary endpoints included the number and location of OASGs and their standard uptake value (SUV) based on FDG-PET/CT data. To highlight factors associated with OASGs, we collected the following data: medical history and anthropometric data (age, sex, body mass index), clinical data (fever, MSK pain), IE characteristics (valve location and type [native or prosthetic; biological or mechanical], the presence of an implantable device, IE classification according to 2015 Duke–ESC criteria [9]), IE origin, complications (death, cardiac surgery, resuscitation), microbiological data (bacteria and blood culture characteristics [number and duration of positivity]), and serum inflammation markers (C-reactive protein [CRP] level and hyperleukocytosis). Results of additional biological or radiological exams to confirm the infectious origin, if performed, were also collected.

### 5.3. Reading and Interpretation of FDG-PET/CT Images

Patients followed a low-carbohydrate diet for at least 24 h to limit myocardial uptake and fasted for 6–12 h before FDG-PET/CT. Images were acquired in 3D mode at 60 min after injection of 4.5 MBq/kg 18F-FDG intravenously by using an FDG-PET/CT system at the nuclear medicine department of Henri Mondor Hospital (a Gemini GXL16 camera, Philips; Da Best, The Netherlands). Transverse PET slices were reconstructed into a 144 × 144 matrix with an iterative 3D reconstruction algorithm with use of the software LOR-RAMLA Body. Low-dose CT was used for PET attenuation correction and for localization of hypermetabolic lesions. FDG-PET/CT was interpreted by three nuclear medicine experts (LL, BE, and MA) blinded to clinical data. OASGs were visually defined by intense and/or heterogeneous uptake involving bone or osteoarticular surfaces and underlying scannographic lesions (bone erosion, abscess, etc.). In the presence of joint prothesis or osteosynthetic surgical material, fixation abnormalities should persist on uncorrected attenuation slices.

We excluded MSK lesions related to a degenerative origin defined according to FDG-PET/CT analysis by a slight or moderate FDG uptake and the presence of degenerative lesions in CT such as subchondral bone changes (sclerosis or cyst formation), osteophytes, and/or joint space narrowing. In case of uncertainty about the infectious or degenerative origin, the OASG diagnosis was rejected.

### 5.4. Statistical Analysis

Patients with OASGs are described according to their main demographic, clinical, and biological characteristics. Categorical data are reported as percentage (number) and quantitative data as median (interquartile range) or mean [standard deviation (SD)]. The association between OASGs and potential factors was estimated by univariate and multivariate analyses. Variables associated with the outcome at *p* ≤ 0.20 in univariate analysis but also age and sex were included in a multivariate model. Variables highly correlated with each other were excluded from the multivariate analyses to ensure independence of identified factors. Multivariate analysis involved logistic regression, estimating adjusted odds ratios (aORs), and 95% confidence intervals (95% CIs). To ensure the robustness of our results, we performed the following sensitivity analyses: (1) restricted to patients with definite or possible IE according to the 2015 Duke–ESC criteria; (2) restricted to patients with only definite IE; and (3) restricted to patients with at least one positive blood culture. We also performed pre-specified subgroup analyses in patients (1) without MSK pain; (2) without an articular portal of entry; or (3) with a native valve.

*p* ≤ 0.05 was considered statistically significant. All statistical analyses were performed with RStudio v3.6.3.

## 6. Results

### 6.1. Description of the Population

**Figure 1:**
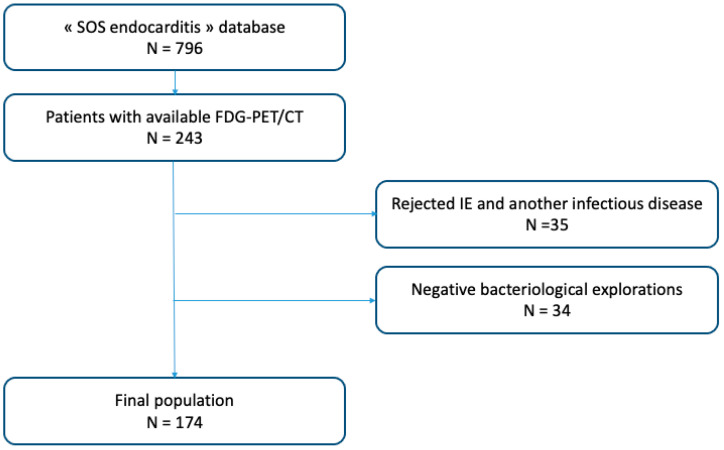
Flowchart of the study.

Among the 796 patients included in the “SOS endocarditis” cohort between January 2015 and December 2020, 243 had FDG-PET/CT images. We excluded patients for whom the diagnosis of IE was not retained in favor of another infectious diagnosis (*n* = 35) and patients for whom all bacteriological samples were finally negative (*n* = 34). Therefore, 174 patients were included in our analysis (Figure 1). Among them, 149 patients had definite (*n* = 110) or possible (*n* = 39) IE according to the 2015 Duke–ESC criteria. The remaining 25 patients had clinically isolated bacteremia without IE. Their mean age was 72.3 ± 13.1 years, and 66.7% were men (Table 1).

**Figure 2:**
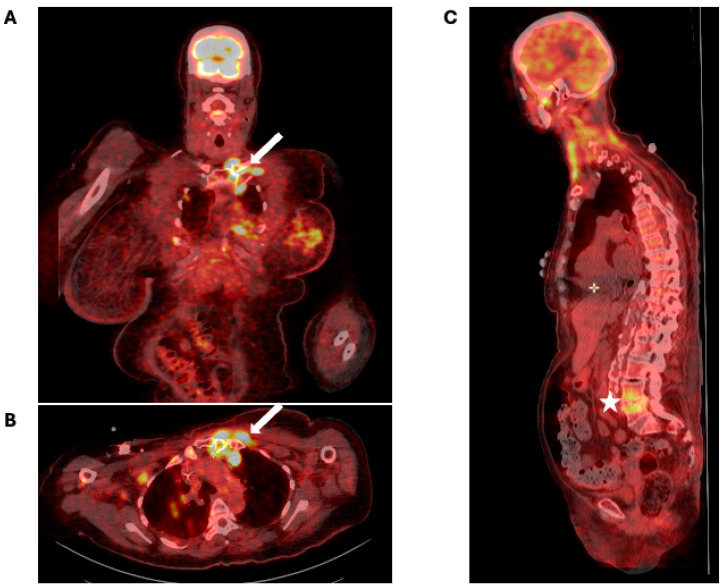
Sternoclavicular and spine OASGs detected by FDG-PET/CT. (**A**,**B**): Arthritis of the left sternoclavicular joint (white arrow; (**A**): coronal section, (**B**): axial section); (**C**): spondylodiscitis at the L4–L5 level (white star).

According to FDG-PET/CT data, 72 distinct OASG locations were identified in 48 patients (27.6%), including 14 patients (29.2%) with multiple OASG locations (Figure 2). A total of 21 patients (43.8%) had OASGs in the spine, mainly located in the lumbar spine (12/21, 57.1%). Nine and one patients had thoracic and cervical OASGs, respectively. Only one patient had multiple spine OASG locations, affecting both the cervical and lumbar spine. Other common locations were glenohumeral and coxofemoral joints (nine patients [18.8%] for each location) and sternoclavicular joint for seven patients (14.6%). Overall, 21 of the 48 patients (43.8%) did not describe any MSK pain located in the site of FDG-PET/CT hypermetabolism. The mean SUV for the OASGs was 6.8 ± 3.0; with a maximum of 29. Forty-one patients (85.4%) had definite or possible IE. The main clinical and biological characteristics of patients are in Table 1.

Among the 48 patients with OASGs, 36 (75.0%) had 6 weeks of antibiotic therapy (from the time of germ identification, usually by blood cultures, and not necessarily at the time of diagnosis of the OASG), and 6 patients (12.5%) had 28 days of antibiotic therapy, including 2 with spinal fixation evoking spondylodiscitis and so should have received 6 weeks of antibiotics according to recommendations. Six patients received only 14 days of antibiotics for isolated bacteremia (thoracic spine fixation, coxofemoral fixation, glenohumeral fixation, sternoclavicular fixation, sternal fixation, and periprosthetic fixation around hip prosthesis, *n* = 1 for each localization).

The antibiotics administered were aminoglycoside (gentamycin or amikacin) in 37 patients (77.1%), amoxicillin in 25 (52.1%), rifampicin in 15 (31.3%), a third-generation cephalosporin (cefotaxime or ceftriaxone) in 14 (29.2%), and a first-generation cephalosporin (cefazolin) in 10 (20.8%). All patients with definite or possible IE received initial dual intravenous therapy.

### 6.2. Predictors of Osteoarticular Septic Grafting

OASGs were not significantly associated with anthropometric data, medical history or comorbidities. Although not significant, the presence of OASGs was associated with obesity (OR = 1.19 [1.00–1.41], *p* = 0.052) and decreased with diabetes mellitus (OR = 0.88 [0.76–1.01], *p* = 0.060) (Table 2). OASGs were associated with the presence of MSK pain (OR = 1.53, *p* < 0.001). OASGs were not associated with the classification of IE (possible/definite) or type (native or prosthetic) of infected valve, but there was a statistical trend for a negative association between OASGs and the presence of mechanical prosthetic valves (*p* = 0.060). OASGs were not associated with the presence of a pacemaker or implantable cardioverter defibrillator (OR = 1.03, *p* = 0.653). Concerning the IE location, OASGs were only associated with tricuspid valve involvement (OR = 1.70, *p* < 0.001).

The identification of Staphylococcus aureus as the causative agent was significantly associated with the presence of OASGs (OR = 1.18, *p* = 0.047). This association was not found for any other infectious agent. Positivity of blood cultures but also their number and positivity time were significantly associated with the presence of OASGs (OR = 1.17, 1.03, and 1.00; *p* = 0.011, 0.002, and 0.015, respectively). The articular portal of entry was significantly associated with the presence of OASGs (OR = 1.63, *p* = 0.023). OASG detection was not associated with CRP level. However, it was associated with the presence of hyperleukocytosis (OR = 1.18, *p* = 0.014) (Table 2).

Age, sex, and all associated factors in univariate analysis (articular portal of entry, hyperleukocytosis, diabetes mellitus, obesity, MSK pain, tricuspid valve involvement, mechanical prosthetic valve, Staphylococcus aureus infection, and the number of positive blood cultures) were included in the multivariate analysis, except for variables that were highly correlated with each other. After adjustments, OASGs remained independently associated with MSK pain and tricuspid valve involvement. A trend toward an association with hyperleukocytosis and with the number of positive blood cultures was also found (OR = 1.12, *p* = 0.059 and 1.02, *p* = 0.053, respectively) (Table 3).

Sensitivity analysis restricted to patients with definite or possible IE, to patients with definite IE, and to patients with at least one positive blood culture provided the same results (Supplementary Tables S1–S3).

In the subgroup of the 139 patients without MSK, the only factor significantly associated with the presence of OASGs was tricuspid valve involvement (OR = 2.18, *p* < 0.001) (Supplementary Table S4).

Among the 169 patients with a non-articular portal of entry, the presence of an OASG was associated with the presence of MSK pain, tricuspid valve involvement, and the number of positive blood cultures (OR = 1.43, 1.50, 1.03; *p* < 0.001, *p* = 0.004, *p* = 0.006, respectively). It was associated but not significantly with hyperleukocytosis but also obesity (OR = 1.12, 1.17; *p* = 0.073, *p* = 0.068) (Supplementary Table S5).

Finally, among the 81 patients with a native valve, the presence of an OASG was associated with MSK pain and hyperleukocytosis (OR = 1.34, 1.24; *p* = 0.011, *p* = 0.031) as well as tricuspid valve involvement although not significantly (OR = 1.37, *p* = 0.085) (Supplementary Table S6).

## 7. Discussion

Our study is the first to assess the prevalence of OASGs revealed by FDG-PET/CT in a large cohort of patients with suspected IE and to identify its predictors. In the 174 patients, the diagnosis of IE was finally confirmed for most patients (*n* = 149), but isolated bacteremia without IE was retained for 25 patients. We identified 72 distinct OASG locations in 27.6% of patients; 14 patients had several OASG locations. Almost half of OASG locations were asymptomatic. In multivariate analysis, the presence of an OASG was associated with MSK pain located at the site of the OASG and tricuspid valve involvement.

As compared with previous published cohorts of IE patients, our patients were older, with a mean age of 72.3 years. The prevalence of native valve IE in our cohort was lower than in the literature (64–67%), but the prevalence of IE in prosthetic heart valves was higher (57.0% vs. 25–34%) as was that of implantable cardiac devices (29.5% vs. 7–11%) [13,14]. These differences were probably due to selection bias. Indeed, until the publication of the Duke–ISCVID and 2023 Duke–ESC criteria [2,3], FDG-PET/CT was mainly performed in patients with suspected IE with a prosthetic heart valve, which may explain their overrepresentation in our cohort. The origin of the infection was identified in only 54.3% of our patients, which is lower than in the literature (almost 76% of cases) [15]. The infections were mainly cutaneous (20.2%), digestive (15.6%), dental (8.1%), and urological (4.0%). Only 2.3% of patients had an infection of articular origin. This finding was globally consistent with the literature, except for the dental portal of entry, which was more frequent [15].

We found an OASG rate of 27.6%, which is consistent with results provided by a recent study assessing FDG-PET/CT data [10], and significantly higher than the 15% initially described in this population [16]. The discrepancy with the prevalence found in our first study (19.1%) [12] is probably explained by a significant difference in the number of patients included, which was much higher in the second study (174 vs. 89). On the other hand, the population assessed was not exactly the same, since in the first study, we only included patients with IE, whereas in the second we included any patient with suspected IE, even if it was not confirmed. Of note, 7 of these 48 patients had no IE but rather clinically isolated bacteremia. In all, 27 patients (56.3%) did not describe any MSK symptoms, and therefore the OASG could not have been diagnosed without FDG-PET/CT.

We did not observe any impact of OASG detection by FDG-PET/CT on disease severity according to length of hospital stay, transfer to intensive care, need for cardiac surgery, recurrence, or mortality. However, our study was not designed to demonstrate the value of FDG-PET/CT in improving the evolutionary profile of the infection, given the absence of a control group and standardized follow-up. Indeed, no systematic follow-up of the cohort was planned. On the other hand, if the patient returned to our center after the infectious episode, we were able to access their medical data to monitor disease evolution and particularly the occurrence of infectious recurrence. Other studies have shown that FDG-PET/CT could reduce the risk of IE relapse by 50% [17], notably by the detection of secondary material infections (implantable cardioverter defibrillator, orthopedic prosthesis, and venous line).

FDG-PET/CT results may influence antibiotic management, such as preferentially selecting an antibiotic with better bone diffusion [18] or modifying the dose and/or duration even if the current trend is to shorten antibiotic therapy in joint and bone infections, converging with that recommended for the main IE (4–6 weeks in most cases) [9,19]. Interestingly, in our study, the duration of antibiotic treatment was too short for eight patients, given the associated bone infection discovered retrospectively after re-reading FDG-PET/CT images, and at least one case had a recurrence of IE several years later. In addition, certain OASGs such as spondylodiscitis may require orthopedic management, either by surgery or immobilization in order to prevent neurological or orthopedic complication. Similarly, systematic surgery is required for an OASG located on a joint prosthesis. Finally, the sooner these OASGs are identified, the sooner specific treatment can be initiated, limiting the potential destruction of joints or discs that leads to chronic pain and disability for patients. We could also expect a financial interest in performing FDG-PET/CT more systematically. Indeed, although this examination is more costly than other imaging procedures, it could reduce the length of hospital stays, readmissions, and need for additional treatment by detecting potentially serious complications at an early stage.

Highlighting factors associated with the presence of OASGs can help physicians identify situations in which FDG-PET/CT should be performed. Age, sex, and comorbidities did not seem to be associated with OASGs.

In multivariate analysis, a significant association was found between OASGs and tricuspid valve involvement in patients with IE. Interestingly, a previous study had shown a high frequency of osteoarticular septic embolism (29.0%) in patients with pacing-lead IE [10]. These results may suggest a link between OASGs and infection localized to the right heart. However, this result should be interpreted with caution because only nine patients had a tricuspid valve infection. Nevertheless, the association between these two conditions remains highly significant in multivariate analysis performed in the whole population but also in various sensitivity or subgroup analyses (supplementary tables), which reinforces these results. On the other hand, we cannot exclude the possibility that IE located in tricuspid valve is a consequence of an osteoarticular infection that is erroneously considered as an IE-related OASG. Apart from the tricuspid location, we found no other risk factors associated with the type of cardiac lesion. Specifically, we found no association between OASGs and prosthetic valves or implantable devices.

*Staphylococcus aureus* was the main bacterium isolated (37 patients), followed by *Enterococcus faecalis* (33 patients), *Streptococcus galoliticus* (12 patients), and *Staphylococcus epidermidis* (10 patients), and was significantly associated with OASGs in univariate analysis. However, this result was not confirmed in multivariate analysis. Nevertheless, the identification of *Staphylococcus aureus* as the causative organism of suspected IE should lead to greater caution regarding the risk of associated OASGs. In our previous study, we found an association between *Enterococcus faecalis*-related IE and the presence of OASGs, which was no longer found in this new analysis. This discrepancy may be due to a bias induced by the low number of patients included in the first study (89 vs. 174).

OASGs were associated with blood culture positivity as well as the time to positivity and number of positive blood cultures reflecting persistent bacteriemia in univariate analysis. In multivariate analysis, they were associated but not significantly with the number of positive blood cultures (*p* = 0.053) in the entire cohort and significantly associated in the subgroup of patients without an articular portal of entry (*p* = 0.006).

No association was found between the presence of OASGs and the origin of infection, except for the articular portal of entry in univariate analysis but not in multivariate analysis (*p* = 0.228). However, these articular portals of entry (three hip prothesis infections and one spondylodiscitis), were considered as OASGs according to FDG-PET/CT analysis, which biases the results. To minimize this phenomenon, we performed a subgroup analysis excluding patients with an articular portal of entry. As for the entire cohort, this subgroup exhibited an association between OASG and MSK pain, tricuspid valve involvement, and the number of positive blood cultures.

This study has several strengths. First, it is the largest retrospective re-analysis of patients with suspected IE who underwent FDG-PET/CT. In addition, it was the largest to specifically focus on OASGs. Moreover, we performed a sensitivity analysis of patients with confirmed IE as well as patients with at least one positive blood culture, which confirmed the association between the presence of MSK pain or tricuspid valve involvement and the occurrence of OASGs, which reinforced the robustness of the results.

However, this study has also some limitations. The first one is that our data were extracted from a cardiology database, in which some rheumatological relevant information was not systematically collected, such as previous rheumatic diseases, precise descriptions of MSK pain, as well as the presence of local inflammatory symptoms such as joint effusion and specific rheumatological management. We cannot rule out the possibility that certain inflammatory rheumatologic diseases that cause non-septic FDG-PET/CT fixation may have favored IE, especially in the presence of immunosuppressive therapy, and thus biased the results. However, these diseases are rare, and it is highly unlikely that this would have significantly impacted our results. Second, we were unable to ascertain the initial origin of infection for patients with IE and OASGs. Indeed, some OASG locations might not have resulted from a septic embolus from the heart but rather were the origin of the IE. These elements demonstrate the need to initially conduct the most thorough and precise clinical examination. Finally, we cannot exclude the possibility that the selection bias related to the fact that we included all patients with a suspected IE may have had an impact on our data. Indeed, in this larger population, there may be slightly more patients in whom the OASG was in fact the primary source of infection. Similarly, we may also assume that the characteristics of patients who underwent FDG-PET/CT (mainly patients with a prosthetic valves or foreign cardiac device) may also have influenced our results. However, these factors were not associated with the occurrence of OASGs in univariate analysis. In addition, our findings regarding the predictive factors for OASGs were strengthened by their consistency in multiple sensitivity and subgroup analyses.

## 8. Conclusions

FDG-PET/CT is a great help in identifying multiple infectious locations. A total of 27.6% of patients hospitalized for suspected IE presented an OASG (mainly involving the spine) according to FDG-PET/CT interpretation, but almost half of them remained asymptomatic. MSK pain and tricuspid valve infection were the main factors associated with OASGs. Thus, we suggest rigorous and systematic MSK clinical evaluation of all patients presenting suspected IE.

Based on the 2023 ESC guidelines suggesting that PET-FDG/CT could be considered for screening peripheral lesions in asymptomatic patients, we believe it could be particularly useful in patients with tricuspid endocarditis or a sustained bacteremia to detect an asymptomatic OASG. Thus, antibiotic management could be adjusted and orthopedic management initiated, which could improve the clinical outcome and prevent septic recurrence or MSK sequelae. FDG-PET/CT could also be performed after the identification of a first OASG by another examination to eliminate a second OASG located at a distance, especially on the spine.

## Figures and Tables

**Table 1:** Description of the population with suspected infective endocarditis (*n* = 174 unless indicated).

	% (*n*) or Mean ± SD
Age, years	72.3 ± 13.1
Sex (male)	66.7 (116)
Diabetes mellitus	31.6 (55)
Hypertension	66.7 (116)
BMI	25.67 ± 5.7
Obesity	17.9 (31)
Immunodepression	41.4 (72)
Cancer	20.1 (35)
IV drug addiction	1.1 (2)
**Cardiovascular condition**	
Heart disease	37.4 (65)
Native valve	46.6 (81)
Prosthetic valve	53.4 (93)
Biologic	37.4 (65)
Mechanical	14.4 (25)
Missing data	1.7 (3)
**Characteristics of infection**	
Definite or possible endocarditis	85.6 (149)
Endocarditis certainty according to Duke criteria (*n* = 149)	
Definite	63.2 (110)
Possible	22.4 (39)
Rejected	14.4 (25)
Bacteremia without endocarditis	14.4 (25)
Location of endocarditis (*n* = 149)	
Left heart	73.2 (109)
Aortic	52.3 (78)
Mitral	24.8 (37)
Right heart	6.7 (10)
Pulmonary	0 (0)
Tricuspid	5.2 (9)
Missing data	0.7 (1)
Pacemaker	25.5 (38)
Implantable cardioverter defibrillator	8.1 (12)
**Clinical data**	
Musculoskeletal pain	19.7 (34)
Fever	81.5 (141)
**Laboratory analysis**	
C-reactive protein (mg/L)	95.0 ± 81.5
Hyperleukocytosis	45.4 (79)
**Microbiological data**	
Positive blood cultures	84.5 (147)
All cocci infection	79.3 (138)
All *Staphylococcus* species	32.8 (57)
*S. aureus*	21.3 (37)
*S. epidermidis*	5.7 (10)
Other *Staphylococcus* species	5.7 (10)
All *Streptococcus* species	25.9 (45)
*S. gallolyticus*	6.9 (12)
Gram-negative bacillus infection	14.4 (25)
*Enterococcus faecalis*	18.9 (33)
Other bacterial infection	16.1 (28)
Unknown bacterial infection	7.5 (13)
**Portal of entry**	
Skin	20.2 (35)
Dental	8.1 (14)
Articular	2.3 (4)
Unknown	45.7 (79)
Digestive	15.6 (27)
Urologic	4.0 (7)
Ear, nose, and throat	2.3 (4)
**OASG**	27.6 (48)
Spine	12.1 (21)
Sternoclavicular	4.0 (7)
Coxofemoral	5.2 (9)
Glenohumeral	5.2 (9)
Sacroiliac	1.1 (2)
**Severity/complications**	
Length of hospitalization	23.8 ± 17.6
Death	16.2 (28)
Transfer to intensive care unit	36.4 (63)
Relapse	2.9 (5)

SD: standard deviation; IV: intravenous; OASG: osteoarticular septic graft.

**Table 2:** Univariate analysis of factors associated with osteoarticular septic graft.

	OASG+ % (*n*) or Mean ± SD *n* = 48	OASG− % (*n*) or Mean ± SD *n* = 126	OR (95% CI)	*p*-Value
Age	74.08 ± 12.9	71.4 ± 13.0	1.00 (1.00–1.01)	0.151
Sex (male)	56.0 (27)	70.6 (89)	0.88 (0.76–1.01)	0.073
Hypertension	70.0 (34)	65.1 (82)	1.05 (0.91–1.21)	0.475
Diabetes mellitus	20.0 (10)	35.7 (45)	0.88 (0.76–1.01)	0.060
BMI (kg/m^2^)	26.0 ± 6.0	25.3 ± 5.4	1.01 (1.00–1.02)	0.155
Obesity	27.0 (13)	14.4 (18)	1.19 (1.00–1.41)	0.052
Cancer	22.0 (11)	19.1 (24)	1.05 (0.89–1.24)	0.572
Immunodepression	41.0 (20)	41.3 (52)	1.00 (0.88–1.15)	0.962
Pre-existing cardiac disease	35.0 (17)	38.1 (48)	0.98 (0.85–1.12)	0.746
**Musculoskeletal pain**	**43.0 (21)**	**10.4 (13)**	**1.53 (1.31–1.79)**	**<0.0001**
Fever	81.0 (39)	81.6 (102)	1.00 (0.84–1.18)	0.958
Confirmed IE	85.0 (41)	85.7 (108)	1.00 (0.82–1.20)	0.960
Definite IE	64.0 (31)	62.7 (79)	1.02 (0.89–1.17)	0.819
Possible IE	20.0 (10)	23.0 (29)	0.98 (0.83–1.15)	0.759
Rejected IE	14.0 (7)	14.3 (18)	1.00 (0.83–1.22)	0.960
Left heart	58.0 (28)	64.3 (81)	0.95 (0.83–1.09)	0.471
Aortic	43.0 (21)	45.2 (57)	0.99 (0.86–1.13)	0.861
Mitral	18.8 (9)	22.2 (28)	0.96 (0.82–1.13)	0.619
**Tricuspid**	**14.6 (7)**	**1.6 (2)**	**1.70 (1.27–2.27)**	**0.0005**
PM or ICD	31.3 (15)	27.8 (35)	1.03 (0.89–1.20)	0.653
Native valve	50.0 (24)	45.2 (57)	1.04 (0.91–1.19)	0.576
Prosthetic valve	50.0 (24)	54.8 (69)	0.96 (0.84–1.10)	0.576
Biologic	41.7 (20)	35.7 (45)	1.05 (0.92–1.21)	0.471
Mechanical	8.3 (4)	19.0 (24)	0.83 (0.69–1.01)	0.060
**Portal of entry**				
**Articular**	**6.4 (3)**	**0.8 (1)**	**1.63 (1.05–2.53)**	**0.023**
Skin	19.2 (9)	20.6 (26)	0.98 (0.83–1.16)	0.830
Dental	6.4 (3)	8.7 (11)	0.94 (0.74–1.20)	0.617
Digestive	21.3 (10)	13.5 (17)	1.12 (0.94–1.35)	0.212
Ear, nose, and throat	0	3.2 (4)	0.75 (0.49–1.18)	0.219
Urological	2.1 (1)	4.8 (6)	0.87 (0.62–1.23)	0.437
**Main bacterial species**				
All cocci infection	85.4 (41)	77.0 (97)	1.11 (0.94–1.31)	0.222
All *Staphylococcus* species	43.8 (21)	28.6 (36)	1.15 (1.00–1.32)	0.057
***S. aureus***	**31.3 (15)**	**17.5 (22)**	**1.18 (1.00–1.39)**	**0.047**
*S. epidermidis*	4.2 (2)	6.4 (8)	0.92 (0.69–1.23)	0.583
All *Streptococcus* species infection	22.9 (11)	27.0 (34)	0.96 (0.82–1.12)	0.586
*S. galloliticcus*	8.3 (4)	6.4 (8)	1.06 (0.82–1.38)	0.647
*Enterococcus faecalis*	16.7 (8)	19.8 (25)	0.81 (0.32–1.79)	0.633
All bacilli infection	12.5 (6)	15.1 (19)	0.96 (0.79–1.16)	0.667
*Escherichia coli*	4.2 (2)	0.8 (1)	1.49 (0.89–2.48)	0.128
Other bacteria	14.6 (7)	16.7 (21)	0.97 (0.81–1.16)	0.740
No bacteria	4.2 (2)	8.7 (11)	1.11 (0.93–1.34)	0.254
**Positive blood cultures**	**89.6 (43)**	**82.5 (104)**	**1.17 (1.04–1.31)**	**0.011**
**Number of blood cultures**	**5 ± 5.3**	**3.0 ± 3.0**	**1.03 (1.01–1.05)**	**0.002**
**Duration of positivity**	**101.3 ± 104.9**	**63.1 ± 71.2**	**1.00 (1.00–1.00)**	**0.015**
**Hyperleukocytosis**	**60.4 (29)**	**39.7 (50)**	**1.18 (1.04–1.35)**	**0.014**
C-reactive protein (mg/L)	110.0 ± 102.9	89.3 ± 71.4	1.00 (1.00–1.00)	0.135

OASG: osteoarticular septic graft. OR: odds ratio. 95% CI: 95% confidence interval. BMI: body mass index. IE: infective endocarditis. PM: pacemaker. ICD: implantable cardioverter defibrillator. OR: odds ratio. 95% CI: 95% confidence interval. The bold characters indicate significant results.

**Table 3:** Multivariate analysis of factors associated with osteoarticular septic graft.

	aOR (95% CI)	*p*-Value
Sex (male)	0.95 (0.84–1.38)	0.472
Age	1.00 (1.00–1.01)	0.328
Articular portal of entry	1.27 (0.86–1.89)	0.228
Hyperleukocytosis	1.12 (1.00–1.27)	0.059
Diabetes mellitus	0.90 (0.79–1.02)	0.100
Obesity	1.15 (0.97–1.36)	0.110
**Musculoskeletal pain**	**1.42 (1.23–1.65)**	**<0.0001**
**Tricuspid valve**	**1.51 (1.16–1.97)**	**0.002**
Mechanical prosthetic valve	0.88 (0.74–1.05)	0.159
*Staphylococcus aureus* infection	1.04 (0.89–1.22)	0.581
Number of positive blood cultures	1.02 (1.00–1.04)	0.053

aOR: adjusted odds ratio. 95% CI: 95% confidence interval. The bold characters indicate significant results.

## Data Availability

The data underlying this article will be shared upon reasonable request to the corresponding author.

## Supplementary Materials

The following supporting information can be downloaded at: https://www.mdpi.com/article/10.3390/jcm13185419/s1, Table S1: Multivariate analysis of factors associated with osteoarticular septic graft in patients with either definite or possible infective endocarditis; Table S2: Multivariate analysis of factors associated with osteoarticular septic graft in patients with definite infective endocarditis; Table S3: Multivariate analysis of factors associated with osteoarticular septic graft in patients with at least one positive blood culture; Table S4: Multivariate analysis of factors associated with osteoarticular septic graft in patients without musculoskeletal pain; Table S5: Multivariate analysis of factors associated with osteoarticular septic graft in patients without an articular portal of entry; Table S6: Multivariate analysis of factors associated with osteoarticular septic graft in patients with a native valve.
