# Supplementary material for: Value of Fluorodeoxyglucose Positron Emission Tomography/Computed Tomography in Identifying Osteoarticular Septic Grafts in Suspected Infective Endocarditis: Results from a Large Monocentric Cohort
Source: J Clin Med. 2024 Sep 12;13(18):5419. doi: 10.3390/jcm13185419 (PMC11432416; doi:10.3390/jcm13185419)
Supplement: Supplementary file 1 [file jcm-13-05419-s001.zip › jcm-3187195-supplementary.pdf]

Supplementary Table S1. Multivariate analysis of factors associated with osteoarticular septic graft in patients with either definite or possible infective endocarditis

	OR (95% CI)	p-value
Sex (male)	0.96 (0.84–1.10)	0.535
Age	1.00 (0.99–1.01)	0.754
Articular portal of entry	1.27 (0.86–1.88)	0.234
**Hyperleukocytosis**	**1.15 (1.01–1.31)**	**0.034**
**Diabetes mellitus**	**0.85 (0.74–0.97)**	**0.020**
Obesity	1.09 (0.91–1.31)	0.345
**Musculoskeletal pain**	**1.46 (1.24–1.71)**	**<0.0001**
**Tricuspid valve**	**1.46 (1.12–1.90)**	**0.005**
Mechanical prosthetic valve	0.87 (0.72–1.05)	0.150
Staphylococcus aureus infection	1.11 (0.94–1.31)	0.235
Number of positive blood cultures	1.02 (0.99–1.04)	0.152

OR, odds ratio; 95% CI, 95% confidence interval

Supplementary Table S2. Multivariate analysis of factors associated with osteoarticular septic graft in patients with definite infective endocarditis.

	OR (95% CI)	p-value
Sex (male)	0.91 (0.78–1.07)	0.285
Age	1.00 (0.99–1.07)	0.907
Articular portal of entry	1.22 (0.76–1.95)	0.405
**Hyperleukocytosis**	**1.21 (1.04–1.41)**	**0.016**
**Diabetes mellitus**	**0.80 (0.68–0.94)**	**0.009**
Obesity	1.11 (0.91–1.35)	0.298
**Musculoskeletal pain**	**1.35 (1.12–1.62)**	**0.002**
**Tricuspid valve**	**1.59 (1.15–2.20)**	**0.006**
Mechanical prosthetic valve	0.85 (0.68–1.07)	0.176
Staphylococcus aureus infection	1.10 (0.92–1.31)	0.288
Number of positive blood cultures	1.02 (0.99–1.04)	0.159

OR, odds ratio; 95% CI, 95% confidence interval

Supplementary Table S3. Multivariate analysis of factors associated with osteoarticular septic graft in patients with at least one positive blood culture.

	OR (95% CI)	p-value
Sex (male)	0.97 (0.84–1.12)	0.692
Age	1.00 (1.00–1.00)	0.334
Articular portal of entry	1.30 (0.79–2.13)	0.300
Hyperleukocytosis	1.12 (0.97–1.29)	0.123
Diabetes mellitus	0.89 (0.77–1.03)	0.113
Obesity	1.16 (0.97–1.39)	0.109
**Musculoskeletal pain**	**1.41 (1.18–1.68)**	**<0.001**
**Tricuspid Valve**	**1.73 (1.24–2.43)**	**0.002**
Mechanical prosthetic valve	0.88 (0.71–1.08)	0.220
Staphylococcus aureus infection	1.03 (0.87–1.22)	0.752
Number of positive blood cultures	1.01 (0.99–1.04)	0.251

OR, odds ratio; 95% CI, 95% confidence interval

Supplementary Table S4. Multivariate analysis of factors associated with osteoarticular septic graft in patients without musculoskeletal pain.

	OR (95% CI)	p-value
Sex (male)	0.98 (0.86–1.19)	0.789
Age	1.00 (1.00–1.12)	0.067
Articular portal of entry	1.36 (0.89–2.08)	0.154
Hyperleukocytosis	1.05 (0.93–1.19)	0.408
Diabetes mellitus	0.91 (0.80–1.03)	0.144
Obesity	1.14 (0.96–1.35)	0.127
**Tricuspid valve**	**2.18 (1.58–3.00)**	**<0.0001**
Mechanical prosthetic valve	0.90 (0.76–1.06)	0.200
Staphylococcus aureus infection	1.03 (0.88–1.20)	0.724
Number of blood cultures	1.01 (0.99–1.03)	0.140

OR. odds ratio; 95% CI, 95% confidence interval

Supplementary Table S5. Multivariate analysis of factors associated with osteoarticular septic graft in patients without an articular portal of entry

	OR (95% CI)	p-value
Sex (male)	0.96 (0.84–1.09)	0.541
Age	1.00 (1.00–1.01)	0.363
**Musculoskeletal pain**	**1.43 (1.24–1.66)**	**<0.0001**
Hyperleukocytosis	1.12 (0.99–1.25)	0.073
Diabetes mellitus	0.92 (0.81–1.04)	0.185
Obesity	1.17 (0.99–1.38)	0.068
**Tricuspid valve**	**1.50 (1.14–1.96)**	**0.004**
Mechanical prosthetic valve	0.87 (0.73–1.04)	0.129
Staphylococcus aureus infection	1.05 (0.90–1.23)	0.527
**Number of positive blood cultures**	**1.03 (1.01–1.05)**	**0.006**

OR, odds ratio; 95% CI, 95% confidence interval

Supplementary Table S6. Multivariate analysis of factors associated with osteoarticular septic graft in patients with a native valve.

	OR (95% CI)	p-value
Sex (male)	1.00 (0.82–1.23)	0.972
Age	1.00 (0.99–1.01)	0.884
**Musculoskeletal pain**	**1.34 (1.07–1.66)**	**0.011**
**Hyperleukocytosis**	**1.24 (1.02–1.51)**	**0.031**
Diabetes mellitus	1.04 (0.85–1.28)	0.698
Obesity	1.03 (0.80–1.33)	0.829
Tricuspid valve	1.37 (0.96–1.96)	0.085
Staphylococcus aureus infection	1.12 (0.86–1.45)	0.405
Number of positive blood cultures	1.02 (0.99–1.04)	0.205

OR, odds ratio; 95% CI, 95% confidence interval
